# Before the Erie County Medical Society

**Published:** 1861-08

**Authors:** Austin Flint

**Affiliations:** Professor of the Principles and Practice of Medicine in the Bellevue Hospital Medical College; New York


BUFFALO

### Medical and Surgical Journal

AND REPORTER.

VOL. I. AUGUST, 1861. NO. 1.

### ADDRESS

*Before the Erie County Medical Society, by* Austin Flint, M. D., *Professor of the Principles and Practice of Medicine in the Bellevue Hospital Medical College,*

READ AT THE JUNE MEETING, 1861, BY PROF. EASTMAN

---

Gentlemen of the Erie County Medical Society:

At the Annual Meeting in January, 1858, you did me the honor of electing me President of your Society. As your President it was incumbent on me, on retiring from the Chair at the next annual meeting, to read a valedictory address. In the interim I accepted an appointment in the New Orleans School of Medicine, the duties of which prevented me from being present at the Annual Meeting in 1859. The Society, in consideration of my absence, voted a postponement of the Address to the Semi-Annual Meeting in June of the same year. But before that time arrived I had decided to leave Buffalo, and I was accordingly not able to prepare an address nor to be present at the meeting. The Society had the kindness again to excuse the performance of my duty, and to postpone it until some convenient occasion, which I then hoped would be at the Semi-Annual Meeting in 1860. My duties at the Long Island Hospital, however, prevented my attendance at that meeting. When I may be able to attend a meeting is now uncertain, but I do not forget the obligation which still rests upon me, and I have received too many acts of kindness from members of your Society to desire to avoid a duty, the performance of which will at least be a manifestation of the feelings of gratitude and

W1
BU424

respect with which I hold in remembrance the professional friends with whom I was so many years associated. I shall take pleasure, therefore, in writing a brief Address, which I shall ask some member of the Society to read in my stead at the Annual Meeting in January, 1861.

I have selected as a subject, *My Retrospections of Medical Practice in Buffalo.* This subject suggests itself as not inappropriate, and I trust it will lead to a train of remark not entirely without interest. A much broader subject would be my retrospections of Buffalo, including matters which relate not alone to medicine, but to medical politics and personal experiences in connection with different members of the medical profession. It would not be fitting on this occasion to embrace these in my reminiscences, since they would involve references to events and persons, which might not be acceptable to all. This more comprehensive view of the subject I must reserve for a different occasion, and a date farther removed than the present from the period over which my retrospective history extends. My purpose now is to confine myself to matters pertaining to the practice of medicine, and if I am led to speak of particular events or persons, it will be in a manner to which no one will be likely to take exceptions.

I came to reside in Buffalo in July, 1836. At that time, with a population of about eighteen thousand, there were not far from thirty physicians. By far the greater part of medical practice was in the hands of the regular profession, but at that time, and for some years, the followers of Thomson (of steam, lobelia and red pepper memory,) made something of a show in point of numbers. They were not among the educated and more intelligent classes of society, but some men of influence were believers and advocates of the Thomsonian system. The Legislature had recently granted to the practitioners of this system the right to practice provided they prescribed only indigenous vegetable remedies. Excepting as thus restricted, not only were all irregular practitioners, i. e. all not members of the County Society, legally incompetent to collect dues, but they were liable to be prosecuted and fined. The restriction and disabilities were some years afterward removed, the profession offering no resistance. The decline of the system may be dated from the action of the Legislature which removed all legal obstacles in the way of the practice. As Thomsonism it soon died out, but under the modified form Eclectism the system has continued to retain a few adherents. The Eclectic system of practice, as it is called with a singular disregard of consistency, since the distinctive feature of the system is, that it does *not* allow to its practitioners the freedom of choice among remedies, originated in Thomsonism, but the most positive traits of the latter system were abandoned, viz: the universal application of steam, lobelia and red pepper to the treatment of diseases. In lieu of these the characteristics of the Eclectic system are the repudiation of blood-letting and mercury as remedies.

Homœopathy began to be heard of a few years after I came to Buffalo. It was preceded by animal magnetism, which excited a good deal of public curiosity and interest, and served to prepare the way for the mysteries of Hahnemann. The first person whom I recollect to have heard of as a practitioner of Homœopathy in Buffalo, had been a regular physician, and his application for admission into the Erie County Medical Society was refused on the ground that he professed to be a Homœopathic practitioner. The person referred to gave up the practice after a year or two, and has since become identified with politics. After this, several practitioners appeared in succession, and remained but a short time. However, the system gained slowly in its disciples until they were sufficient to afford business enough for three or four practitioners. Of late years the system could hardly be said to have gained in the number of its friends, but it has acquired new converts enough to nearly, if not quite, make up for defections in its ranks, and thus has remained about stationary. The most successful practitioner of Homœopathy, (I mean as regards the amount of practice,) was a regularly educated physician who was refused admission into the Erie County Medical Society on the score of unprofessional conduct in his relations with a prominent member of the profession.

It would be difficult to continue the systems of practice more completely antagonistical than Thomsonism and veritable Homœopathy. Each is adapted to a different order of mind, and, hence, although the two delusions succeeded each other, the adherents of the one did not transfer their faith to the other. Thomsonism impressed those who were gratified with the idea of reducing medical science, as well as theology and political economy, to principles of common sense, intelligible alike to the unlettered and the learned. That the apostle of this system was an ignorant man, was in its favor. They looked upon the medical profession as an aristocratic body, sustained by legal privileges. Moreover, the demonstrative character of the Thomsonian measures of treatment was suited to persons of this stamp. Homœopathy, on the other hand, secured believers among those who were fond of the mystical. It recommended itself by assuming a kind of transcendental character. It harmonized with certain notions of philosophy, sentiment and refinement. The persons attracted toward it, therefore, were not vulgar worshippers of ignorance enshrined as common sense, but the devotees of the mysterious and marvellous under the name of science. To persons thus constituted, the doctrine that medicinal remedies are potential in infinitesimal quanities, recommended itself the more that it conflicted palpably with common sense.

The evils arising from the prevalence of successive systems of quackery, are very great, but there are not wanting some good results. One of these is the increased attachment to the medical profession, when persons become thoroughly satisfied of the error of opposing the profession. The pride of opinion and the pen of ridicule are powerful motives to prevent an abandonment of any belief which is at variance with public sentiment, but when the mind becomes satisfied that it has been led away by an infatuation, there is little danger of another deviation in the same direction. I have observed that among the warmest and most consistent friends of physicians, are those who have given Thomsonism, Eclecticism or Homœopathy a fair trial, and have had resolution enough to confess the lessons which they have learned from experience.

Of the medical profession in Buffalo in 1836, six members were especially prominent. They constituted the older physicians of the city. They were the leading members of the profession. The cream of medical practice was divided among them, and they were generally called in consultation by the younger members. I do not include in this number Dr. Chapin who, although living at that time, had mostly withdrawn from practice. With one exception, Dr. Marshall, all of this number are now living, and I therefore forbear giving their names. All, however, have retired from practice, one having adopted many years ago other pursuits, and the remainder being incapacitated by disease for the active duties of professional life. These men are fairly entitled to be called the fathers of the medical profession in Buffalo, residing there at the time when it emerged from a small country village into a prominent and populous city. To discuss their respective merits at this time and place would be indecorous, but I may be allowed to speak of them collectively. As representing our profession, they rendered it deserving of respect and confidence. In respect of the character of its representatives, the medical profession at that time did not suffer by a comparison with Law or Divinity, although the two latter professions embraced very able men. Differing in mental constitution, in habits of mind, and in manners, they fraternized with each other in a remarkable manner. It could hardly be otherwise than that some misunderstandings would occasionally arise, but I believe I am correct in saying that there never occurred any interruption of social or professional intercourse.

Professional consultations with each other and with the younger members of the profession, were frequent, and this has ever since been a prominent feature of medical practice in Buffalo. In the great majority of important cases, among the rich and poor, the custom has been for the attending physician to request a consultation with one or more of his professional brethren, and it has been common for two or more physicians to be associated in attendance until the termination of a serious disease is decided. Consultations have been placed on their true basis, viz : on the importance of combined knowledge and judgment in cases involving danger of the loss of life or health ; not on the supposition of want of confidence in the attending physician on the part of patient or friends. An acquaintance with the views and habits of medical men in other places has led me to appreciate the position with regard to this matter, which has been maintained by the physicians of Buffalo. I suspect that in few cases in our country have consultations been so much in vogue as in this city. Consultations have almost always been conducted in a most friendly spirit. During a period of over twenty years, I can recall very few instances in which any troubles have grown out of them ; and on the other hand, irrespective of their benefits in individual cases, I believe that their frequency and a proper appreciation of their objects with the people have done very much toward enhancing public regard for our profession. Not a little credit is due to the medical fathers of Buffalo for leading the way in placing consultations on a proper basis, and it is creditable also to those who followed in their footsteps in this regard, that consultations have remained truly such, instead of becoming occasions for quarrels and intrigues which tend more than any thing else to degrade the profession in the eyes of the community It is but justice to our medical fathers to add, that while from their age and position they exerted a controlling influence, it was by no means uncommon for the younger members to meet them in consultation in their own cases. I can recall numerous instances in which I was associated with them in the relation of a consulting practitioner during the early part of my residence in Buffalo, and treated with as much consideration as if there had not been so great disparity in years ; and this, I doubt not, is the experience of others who were then, like myself, junior practitioners.

Two events in 1845 led to important results as regards their influence on medical practice in Buffalo. One of them was the organization of the *Buffalo Medical Association.* The initiative in this organization was taken by several of the juniors, in conjunction with all of the older practitioners of the city. The objects of the Association were stated by the first President, Dr. Jonah Trowbridge, in his Inaugural Address, to be *First,* "A free and mutual interchange of medical opinions;" and, *Second,* "To cultivate a friendly intercourse, an honorable and gentlemanly deportment, and a strict observance of courtesy towards each other." Nearly all the physicians of the City became at once members of the Association. Subsequently, some ceased to attend the meetings from indifference, and some became estranged from it. The Association, however, was steadily kept up, and a few years since was legally incorporated. I believe that it will not be denied by all who have participated in the meetings of the Association, that the objects, as stated by the first President, were promoted. The free interchange of opinions led to professional improvement, differences and misunderstandings were explained and friendly feelings cultivated. Notwithstanding the differences of opinion and discussions which must of necessity belong to the history of an association open not only to all matters relating to medical science, but to everything pertaining to medical ethics and the professional conduct of its members, there was never any disturbance of the harmony of its meetings during fourteen years, the period which my retrospections of it embrace.

The other event to which I refer was the establishment of the *Buffalo Medical Journal.* I may be excused for mentioning this on the ground that, although I was the editor, and continued the publication of the work for ten years, part of the time as the sole proprietor, and the remainder of the time as a co-proprietor, yet it was commenced at the instance of several of the leading physicians of Buffalo, and the expenses of the first year were guarantied to the publisher by Drs. Sprague, White, Hamilton and myself. The success, however, was such that neither of the gentlemen with whom I was associated in the pecuniary responsibility of the enterprise, were called upon to furnish funds. Another reason why I need not shrink from the mention of this event is, the amount of matter contributed to the pages of the journal by my medical brethren in Buffalo. That the publication of the journal exerted a favorable influence on the medical profession of the City, I may fairly claim, without assuming anything in behalf of its editorship. Numbering among its patrons nearly every practitioner of the City, it contributed to diffuse a knowledge of new developments from scientific research at home and abroad, and of new medical works; it excited emulation and thereby promoted thought, study and observation by means of the contributions from the City and neighborhood.

Another important event was the establishment of the Medical College in 1846. Of the circumstances connected with the origin and early history of this Institution I do not purpose now to speak. The circumstances are still somewhat too recent to be made the subjects of historical record. Such a record, however, I mean to prepare at some future time, if my life be spared. I refer to this event simply as one of those which undoubtedly exerted considerable influence on medical practice in Buffalo. In the first place it influenced in no small measure the habits and aims of those connected with the school as medical teachers. It also afforded opportunities and incitements to others, which would not otherwise have existed. That it tended to raise the *status* of the profession in the City in various ways can not in fairness be denied. On the other hand, that it was made the occasion for professional jealousies and divisions must be admitted. These unpleasant consequences were unavoidable, and they are not peculiar to the origin and early history of the Medical College in Buffalo, but have generally followed similar enterprises in other places.

I may enumerate among the prominent events affecting medical practice in Buffalo during the period over which my retrospections extend, the custom of signalizing the annual meetings of the Erie County Medical Society by social festivities. The members of the Society adjourned to dine together by invitation of the physicians of Buffalo, for the first time, in 1853. After this it became a custom to assemble annually at the festive board, enlivened by toasts, speeches, anecdotes and songs. This convivial intercourse, which never exceeded the bounds of a rational indulgence, contributed to make more close and genial the friendly relations of those who participated in it.

I have spoken of the character of the leading physicians in Buffalo at the time when I came to reside in the City. Twenty years after, all these had ceased to be active members of the profession. Their places were filled by those who twenty years before were the junior practitioners. Of the same number who at the end of twenty years held the same position as regards age, professional position and the amount of practice, all with a single exception, had resided in Buffalo twenty years. In the mean time the population of Buffalo had increased from eighteen to nearly an hundred thousand. The profession have proportionately increased in numbers, consisting here, as elsewhere, of three classes, viz: the senior, the junior, and the young practitioners. I may speak of these, as of the medical fathers of the City, collectively. The majority were united by feelings of friendship, so as to constitute in reality a fraternity. So far from there being a disposition to disparage or overreach each other, I believe I am correct in saying that any one of this number would go first to his medical brethren for sympathy, advice and aid in any difficulty. As regards intelligence, professional attainment and moral qualities, I believe they would not suffer by a comparison with the majority of the members of the medical profession in any other City. Such was and is the medical profession in Buffalo. *Esto perpetua!*

I must apologize, gentlemen, at once for the brevity and the length of this address. Albeit the restriction which I have placed upon the latitude of my subject, it covers a wide field, and I must dismiss the various matters which it embraces with a brief notice of each. My remarks thus far have had reference to medical practice in Buffalo in general terms, as affected by events exerting an influence on the medical profession. I wish to say something having reference more immediately to medical practice. My retrospections show a great change in the current medical opinions with regard to the treatment of diseases. This change, however, is consistent with the progress of medical knowledge, and is perhaps as marked in some other places where the profession have had as much intercourse with each other, and there has existed the same amount of inquiry and discussion. When I came to Buffalo the antiphlogistic treatment of most diseases was in its zenith. Inflammation was considered the great pathological element in practical medicine. All the fevers were regarded as either dependent on local inflammation or as deriving their gravity from inflammatory complications. Most of the neuralgic affections, then perhaps as common as now, were regarded and treated as inflammations. The state of anæmia and other mordid conditions of the blood had hardly began to be studied. The dormant pathological doctrines were based on an exclusive solidism. To support the power of the system by the free use of stimulants and nutriment, under almost all circumstances was stigmatized as *incendiary* treatment, after the favorite expression of Brossais. Bleeding was the "sheet anchor" in the management of most diseases. The chief point for discussion and difference of opinion in individual cases was the extent to which the abstraction of blood should be carried, and whether it should be done by local or general bleeding, or by both combined. Strange as it may now seem, the admirers of Brossais, who trusted to the amount of depletion which could be effected by a fabulous number of leeches and starvation, were regarded by others as given to a censurable inefficiency in their measures of treatment, somewhat as the followers of the expectant method of treatment are now regarded by some. Next to blood-letting, mercury was deemed the most potential agent in subduing inflammation, and a large proportion of patients affected with different diseases were salivated. Indeed, it was pretty well understood that a patient who put himself under the care of a physician incurred the risk of salivation. Emetics and cathartics were used with great profusion, and rigid diet was an almost invariable rule of practice. I need not indicate how far these practical views are in contrast with those which now govern the practice of intelligent practitioners. I do not refer to these by-gone opinions in order to cast any reproach on the knowledge or judgment of the leading practitioners at the time when these opinions were in vogue. They were the current opinions of the day, and by no means peculiar to the City of Buffalo. The able physicians of the city then exemplified in their practice the doctrines held by the most eminent of the medical teachers and writers of the day. The change, indeed, has been great, almost revolutionary. In this fact we have a most gratifying evidence of the progress of the science of medicine. But this reflection should not lead us to regard the present state of the science as perfected. That there has been a great advancement in knowledge is undeniable, and that a corresponding improvement in practice has accomplished much in the saving of life and health cannot be doubted; but there is still an illimitable space for further progress, and should we live to indulge in retrospections at the end of a quarter of a century from the present time, it is probable that the contrast will be as great between medicine then and now, as it is between the present and the past.

It is interesting to me to look backward and trace in my own mind and in the opinions of medical brethren the gradual development of the change which has taken place. It is but justice to our seniors to say that their views of practice underwent considerable modification. But it is no more than justice to say that the junior members of the profession were the active agents in effecting it. In Buffalo, as elsewhere, the older practitioners were disposed to think that the change in practice was in a great measure due to a change having occurred in the human constitution in consequence of which, measures of treatment formerly called for, were no longer borne to the same extent. This notion serves to break the force of the fall from a platform which is no longer tenable, but it is foreign to my purpose to enter into any discussion of it on the present occasion. I deem it but just to the majority of the members of the medical profession in Buffalo during the latter part of the period of my retrospections, to add that, in the transition from the too indiscriminate and excessive use of the so called antiphlogistic and alterative remedies to a better appreciation of the value of soothing and sustaining measures, they have more than kept pace with the progress of the profession at large. As regards the benefit to be derived often from the free use of opium in the treatment of inflammations; the power of alcohol in the essential fevers and all diseases in which the leading indication is to obviate the tendency to death by asthenia, and the importance of efficient alimentation in fulfilling this indication, physicians in Buffalo have been in advance of practical views which have emanated from other sources at home and abroad. They have been already familiar with opinions and modes of practice which have appeared in medical literature as new, and to a large proportion of medical readers probably seemed to be striking novelties. I may refer, as an illustration of this remark, to the publications by the late Dr. Todd, of London, relating especially to the management of fevers and other acute diseases.

A few words respecting the diseases of Buffalo during the period of my residence there.

The first epidemic which occurred, exclusive of the eruptive fevers, was puerperal fever in the spring of 1844. Epidemic erysipelas prevailed to some extent at the same time. These diseases were prevalent at that time in many parts of our country. The epidemic in Buffalo was not severe nor of long duration. A report of nearly all the cases of puerperal fever was prepared by me, in accordance with a resolution of the Erie County Medical Society, and published in the New York Journal of Medicine, then edited by Dr. Charles A. Lee.

The city suffered severely from a visitation of epidemic cholera in the summer of 1849, as was the case when this disease traversed our continent in 1832 and 1834. A pretty full account of the epidemic in 1849 is contained in the number of the Buffalo Medical Journal for the month of November of that year. The visitation was repeated in 1852 and in 1854, attended in both years with considerable mortality. It is worthy of remark that only one member of the medical profession died with the disease and the member referred to, (Dr. Haddock,) was not engaged in medical practice, although an active member of the Board of Health. His death occurred in 1849.

These are the only epidemics of importance (exclusive of Scarlatina, Robeola and Variola,) which prevailed during the twenty-three years that I resided in Buffalo. Cerebro-spinal Meningitis, which formerly prevailed extensively, and with great fatality, in many parts of our country, never made its appearance amongst us. Dysentery prevailed somewhat during the years of the cholera visitations, and in the intermediate years, characterised in certain cases by sero-sanguinolent discharges, collapse and death but the fatality was not large. At no other time has this disease prevailed to an extent or in a form to be considered as an epidemic.

During my residence in Buffalo, I witnessed a change in the indigenous fevers which has been observed in other situations. When I first came to the city, and for several years afterward, the periodical fevers, (Intermitting and Remitting,) were common, and the common continued or typhoid fever, as it had then been studied and described by Louis, was unknown. I can make this statement with positiveness, as I was acquainted with the writings of Louis, and had seen typhoid fever in Boston and the Western part of Massachusetts during my pupilage and the two years which elapsed after my graduation, before my emigration (as it was then considered,) to Buffalo. The first well marked case of typhoid fever which came under my observation in Buffalo, was in 1845, and I regarded it as so much of a curiosity as to ask several of my medical friends to see it, and I reported it in full at a meeting of the Buffalo Medical Association. At this time periodical fevers had greatly decreased. In large proportion of the cases of Intermittents and Remittents, the persons afflicted had contracted these fevers traveling in the Western States during the summer. As these fevers ceased to be indigenous, cases of typhoid fever became more frequent, and during the last half of my residence in Buffalo, more or less of the latter disease was seen every year, but at no time prevailing to much extent.

In 1840, and for several years afterward, cases of typhus, generally known at that time as ship fever, were frequent among the newly arrived immigrants landing at New York, Quebec and Montreal, and arriving at Buffalo on their way to the Western country. The disease prevailed for several years extensively in all the sea port towns which received Irish immigrants. It was never diffused among the inhabitants of Buffalo outside the Poor House and the Hospital of the Sisters of Charity.

In conclusion, gentlemen, I must ask your indulgence in behalf of the fragmentary consideration of the subject which I have selected. I venture to believe that had I given to my retrospection a wider scope, allowed myself ample space, and been under no restraint as regards reference to persons and events—in short, if I had undertaken to write a full record of my life and times in Buffalo, I might have prepared a history which would be interesting to those members of the society at least, with whom I have been for many years closely associated by professional and friendly relations. As it is, I beg you to consider my address, simply as an acquittal of an obligation which has rested upon me, and the choice of my subject as evidence of the disposition on my part to revert to the long period when, my labors and aims in life were connected with the beautiful city which although my destiny has called me from it, I can never cease to consider as a home.

New York, September 1, 1860

